# Why scientific societies should involve more early-career researchers

**DOI:** 10.7554/eLife.60829

**Published:** 2020-09-23

**Authors:** Adriana Bankston, Stephanie M Davis, Elisabeth Moore, Caroline A Niziolek, Vincent Boudreau

**Affiliations:** 1 Board of Directors, Future of ResearchPittsfieldUnited States; 2 ECR leader, American Society for Pharmacology and Experimental TherapeuticsRockvilleUnited States; 3 Center for Information and Study on Clinical Research Participation (CISCRP)BostonUnited States; 4 Department of Communication Sciences and Disorders, University of Wisconsin-MadisonMadisonUnited States; 5 Department of Plant and Microbial Biology, University of California, BerkeleyBerkeleyUnited States; 6 Howard Hughes Medical Institute, University of California, BerkeleyBerkeleyUnited States; 7 Department of Biochemistry and Biophysics, University of California, San FranciscoSan FranciscoUnited States

**Keywords:** research culture, early-career researchers, scientific societies, None

## Abstract

Early-career researchers (ECRs) make up a large portion of the academic workforce. Yet, most leadership positions in scientific societies are held by senior scientists, and ECRs have little to no say over the decisions that will shape the future of research. This article looks at the level of influence ECRs have in 20 scientific societies based in the US and UK, and provides guidelines on how societies can successfully include ECRs in leadership roles.

## Introduction

The majority of scientific research is carried out by graduate students, postdoctoral researchers, and faculty who do not yet have tenure. Collectively known as early-career researchers, this group provides a constant influx of new talent, skills, and ideas (National Institutes of Health, 2011; Heggeness et al., 2017), and is also considerably more diverse in terms of gender and ethnicity than the rest of the research enterprise (Nikaj et al., 2018). However, decisions at universities, funding agencies, publishers, and professional societies tend to be taken by senior researchers. This means that early-career researchers – some of whom will become the senior researchers of the future – are given little or no say in decisions that will shape the future of research (Committee on the Next Generation Initiative, 2018; Alberts et al., 2014).

Scientific societies provide scientists at all career stages with the opportunity to network, to contribute to scientific meetings and conferences, and to participate in professional development activities (Matyas et al., 2017; Ansmann et al., 2014; Kaplan, 2013). These organizations are particularly well-positioned to include early-career researchers (ECRs) in leadership positions for the following reasons:

- Societies are often involved in policy discussions with funders and government agencies.
- Societies provide opportunities for researchers to interact across career stages, institutions and countries.
- Societies are eager to recruit and retain the next generation of leaders in their field.

Some societies already include ECRs in their leadership and have shown that these positions not only elevate the careers of ECRs but also help societies retain members who will eventually become leaders in their respective fields.

## How widespread are ECR leadership positions in scientific societies?

To get an idea of the involvement of ECRs in leadership positions within scientific societies, we identified a list of 20 societies (based in the UK and the US) using the Future of Research Twitter network and mailing list to crowd-source information (Supplementary file 1). From the information collected, we found that less than 2% of the leadership positions available at these societies were held by ECRs. Most of these leadership roles had been established within the last ten years and lasted 2–3 years on average. These positions were primarily held by postdocs, but some were also held by doctoral students and untenured assistant professors.

**Figure 1.**
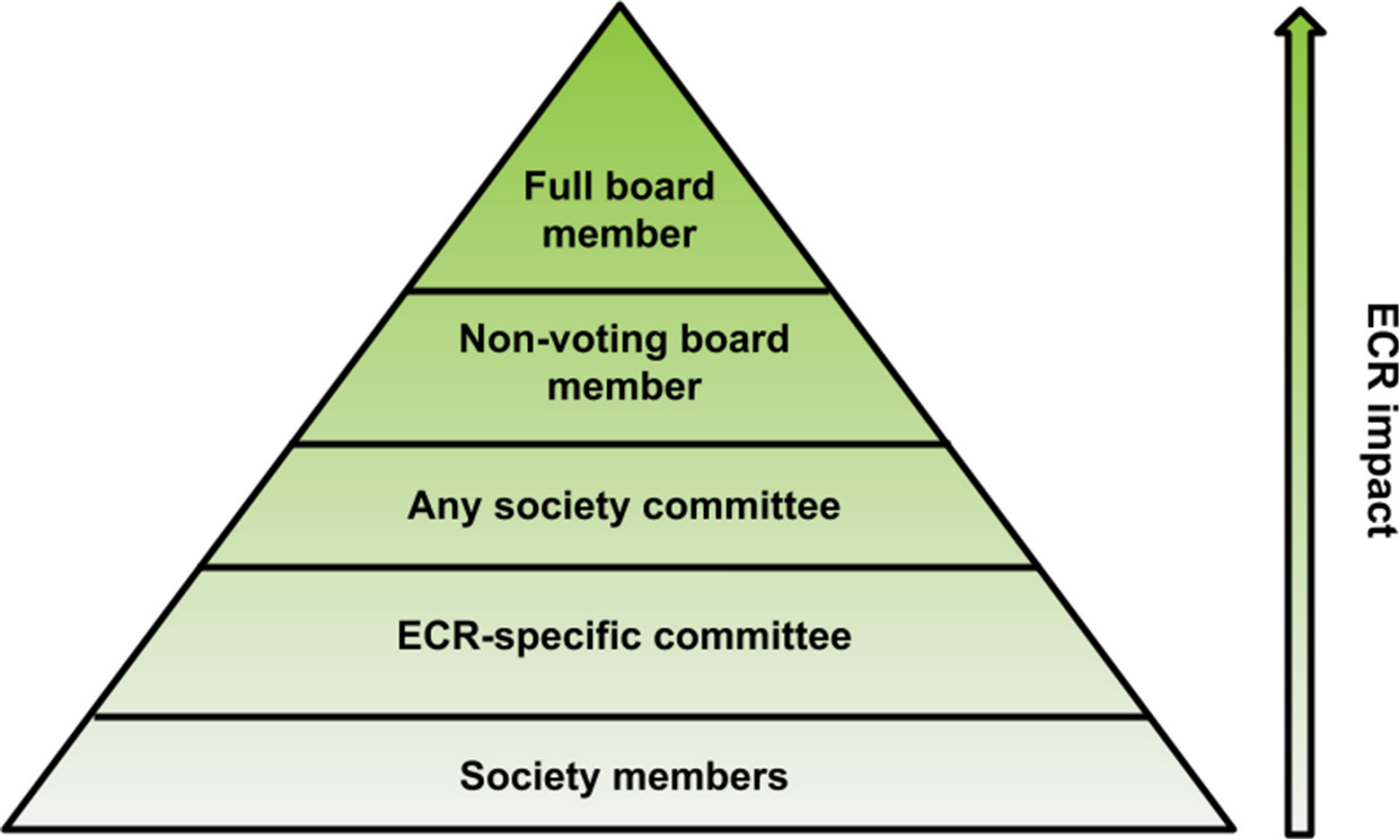
The amount ECRs contribute to a society varies depending on their leadership role. Out of the 20 societies studied, some include ECRs on their Board of Directors, whereas others encourage ECRs to participate in working groups that are specifically for ECRs. Leadership roles that have the most impact on the society, such as being a voting member on the Board of Directors, are less commonly held by ECRs compared to positions that have a lower impact, such as being a member of an ECR specific committee.

ECRs can have varying degrees of responsibility within a society depending on their leadership role (Figure 1). The most impactful way for ECRs to be involved in a society is by allowing them to become full voting members of the Board of Directors or Council. However, in the societies we studied, most ECRs had more moderate responsibilities, ranging from attending and contributing to board meetings where they are involved in discussions but cannot vote (non-voting board members), to serving on society committees or serving on an ECR-specific committee.

For many ECRs looking for leadership opportunities, ECR-specific committees can provide exposure to the society’s structure and policies, allow them to voice their concerns, and contribute to the direction of the society. For example, the American Society for Cell Biology (ASCB) has the Committee for Postdocs and Students (COMPASS) which organizes professional development sessions, runs small outreach grants and other activities at the ASCB annual meeting, publishes weekly blog posts for trainees, and contributes to the ASCB newsletter (American Society for Cell Biology, 2020). The Genetics Society of America (GSA) has several subgroups led by their committee of Early Career Scientists (ECS), which address the challenges of ECRs by providing training in communication, outreach as well as policy and advocacy (Genetics Society of America, 2020a; Genetics Society of America, 2020b).

While ECR-specific committees provide a voice for graduate student and postdoc members, this model of leadership potentially creates silos between the ECRs and senior members of the society. By contrast, assigning ECRs to leadership positions in general committees and on the Board of Directors removes organizational boundaries between ECRs and senior leaders.

**Figure 2.**
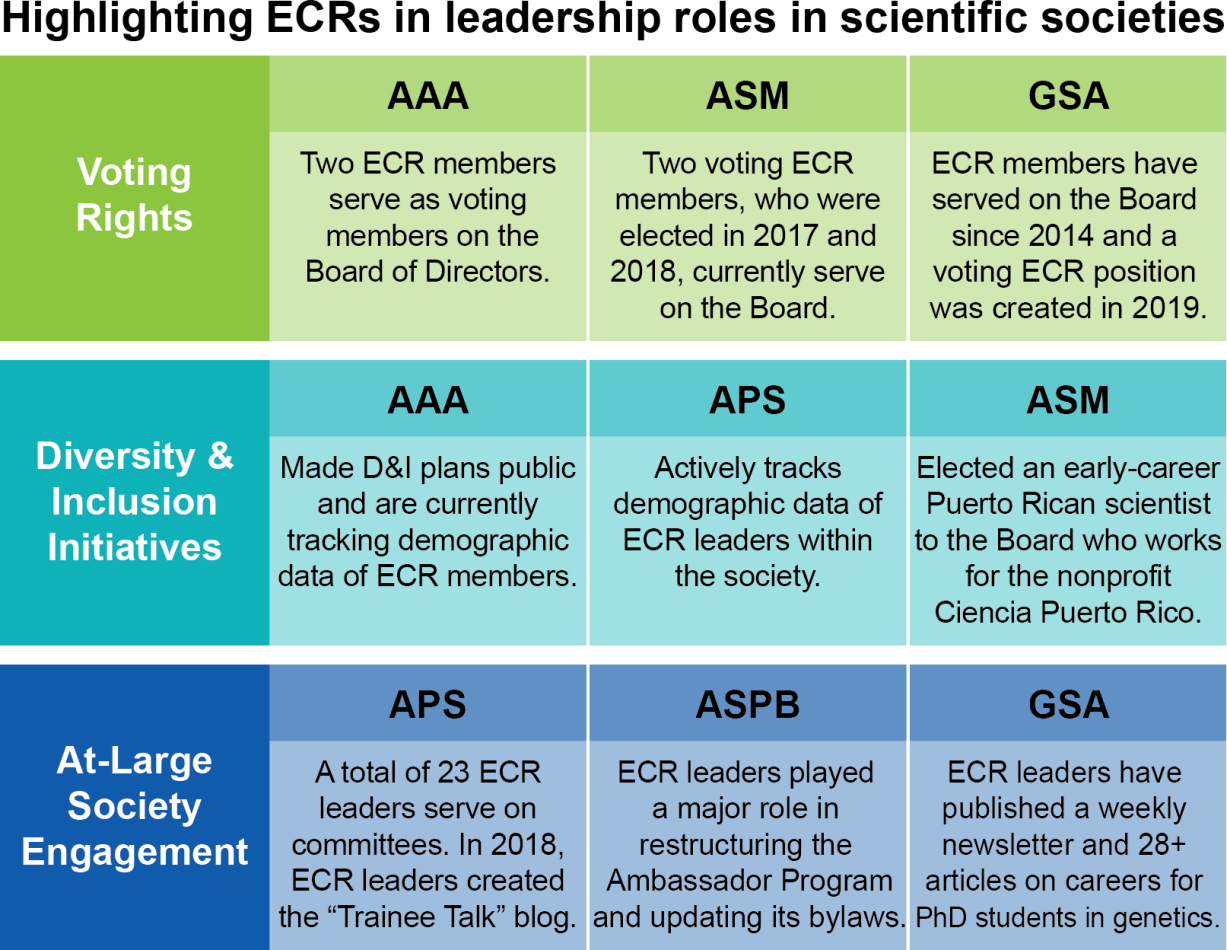
Examples of ECRs in leadership roles in scientific societies. This table highlights societies that have successfully included ECRs in their leadership by giving ECRs voting rights on society boards, including ECRs in their diversity and inclusion initiatives, and more broadly engaging ECRs in different levels of the society. AAA – American Association for Anatomy; ASM – American Society for Microbiology; GSA – Genetics Society of America; APS – American Physiological Society; ASPB – American Society of Plant Biologists.

In the societies we studied, a few stood out regarding the level of influence allotted to their ECR leaders. The American Association for Anatomy (AAA), GSA, and the American Society for Microbiology (ASM) were noteworthy for having multiple ECR members with voting privileges that serve on their Boards or Executive Leadership (Figure 2). The AAA and American Physiological Society (APS) also demonstrated their commitment towards promoting diversity and inclusion by actively tracking the demographic data of ECRs within their leadership and membership (American Association for Anatomy, 2020).

Some societies also include ECR representatives within general committees that are not specifically for ECRs. For example, the APS has two trainee members in their Women in Physiology Committee (American Physiological Society, 2020). The American Society of Plant Biologists (ASPB) has early career members in a number of committees, including the Education Committee, and Science and Policy Committee (American Society of Plant Biologists, 2020a). ECRs also serve on various working groups at the American Society for Pharmacology and Experimental Therapeutics (ASPET), including the Executive Committees for each Division and the Mentoring and Career Development Committee (American Society for Pharmacology and Experimental Therapeutics, 2020a).

Finally, in order to promote outreach to the general membership, GSA (Genetics Society of America, 2020c) and ASPET (American Society for Pharmacology and Experimental Therapeutics, 2020b) publish blogs directed towards ECR members, while ECRs involved in the ASPB help update the bylaws for their Ambassador Program – a self-governing (as of this year) group of students, postdocs, and industry scientists who volunteer to represent ASPB and to communicate its mission to the plant biology community (American Society of Plant Biologists, 2020b).

## Benefits of ECR leadership

To understand more about the leadership positions held by ECRs and what societies gain from their involvement, we reached out to several of the previously identified societies with varying levels of ECR involvement: this ranged from specific committees to designated ECR representatives on the society’s Governing Board. We allowed each society to determine which senior leader and ECR leader would participate in the interview process. Based on the level of responsiveness from each society, we interviewed nine senior society leaders and seven ECR leaders in different positions: this includes one of the authors of this article, Stephanie Davis, who is an ECR leader at ASPET.

**Figure 3.**
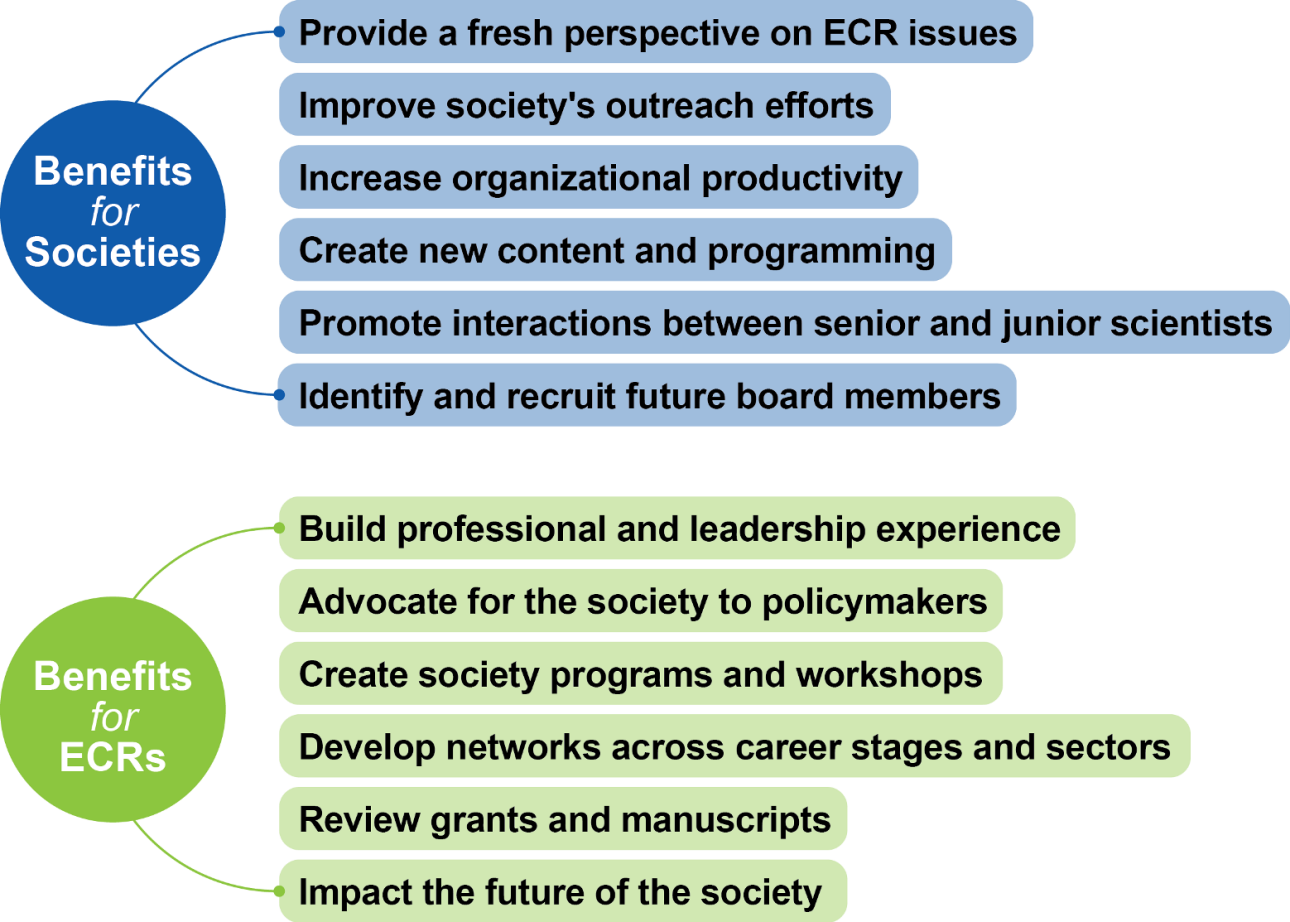
Benefits of ECR leadership positions within scientific societies for both ECRs and societies. The benefits listed in this figure are based on answers given by ECRs and senior society leaders who were interviewed for this article. The interview questions are listed in Supplementary file 2.

Regardless of the type of leadership role, all ECRs reported positive outcomes from these positions, mainly in gaining professional work experience, building professional independence, and contributing to the direction of their research communities (Figure 3). In addition to the professional development and networking benefits provided by societies to ECRs mentioned above, these positions have often led to ECR leader recruitment into different roles within the society or in other organizations, illustrating their significant contributions.

Quoted below are examples of how ECR involvement in scientific society leadership was beneficial for professional development.

 “This position has greatly broadened my professional network, which helped me find my current postdoc position, get reference letters for an NIH fellowship, and provided a crucial support network.”

- Heather Richbourg, Ph.D., Postdoctoral Board Member (AAA)

“I highly value the role that I serve, since it plays a crucial role in advocating for the support of the young scientists across the society. Without proper support and engagement of these young scientists, there is a limited future for the society.”

- Chris Banek, Ph.D., Chair, Trainee Advisory Committee (APS)

Similarly, society executives reported having had positive experiences incorporating ECRs in leadership positions, as illustrated by the quotes below from society leaders at the AAA and ASPB.

“One of the major benefits has been providing the Board with a fresh perspective regarding topics and issues impacting an important sector of our membership… ECRs have brought up multiple issues before the Board that would not have otherwise been considered.”

- Phil Brauer, Ph.D., Past President (2017–2019; AAA)

“The ECR members of the Membership Committee stand out for their active involvement, and we’ve gained a lot by their insights and energy.”

- Jill Deikman, Ph.D., Membership Committee Chair (ASPB)

Both the ECRs and society executives interviewed reported very few negative outcomes of having ECRs in leadership roles. A small number of ECRs said that the work can sometimes be time consuming. While some society leaders reported that mentoring and guiding ECRs in these roles can occasionally present challenges.

Nevertheless, taken together these outcomes indicate that having ECRs in these leadership positions is overall mutually beneficial for both ECRs and societies (Figure 3).

## Recommendations for setting ECRs up for success

Given the relative scarcity of leadership positions reserved for ECRs across the research enterprise and mutually beneficial outcomes for both societies and ECRs, one may ask why so many societies exclude ECRs from leadership roles. Interestingly, most of the queried societies only created leadership positions for ECRs within the last ten years, while fewer than half of these societies allow ECRs to vote in board meetings. This finding suggests that incorporating ECRs into these roles is a developing phenomenon.

**Figure 4.**
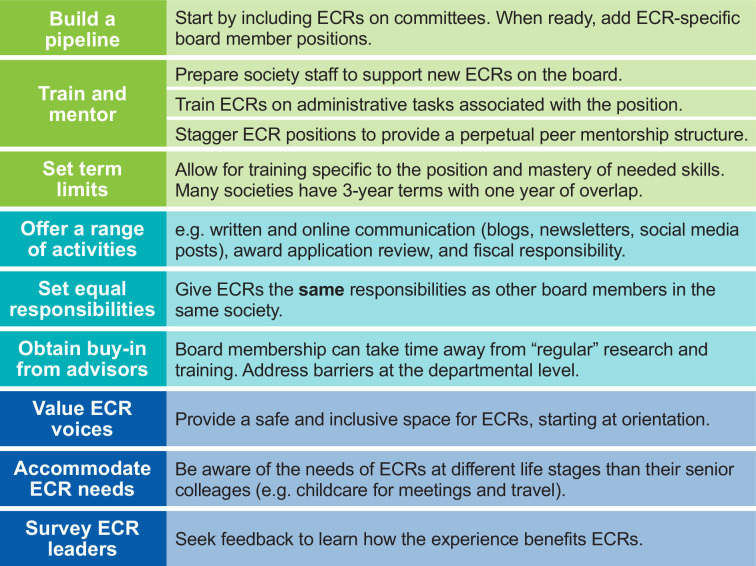
Guidelines for involving ECRs in the leadership and running of scientific societies. Recommendations for establishing leadership positions for ECRs within scientific societies, as recommended by successful programs.

Using the advice provided by society executives, we have compiled a list of guidelines for how scientific societies can successfully establish leadership roles for ECRs (Figure 4). With these guidelines and the benefits outlined in this article, scientific societies should feel empowered to engage more with ECRs and create leadership positions for this group of researchers.

## Conclusion

It is clear to us, based on conducted interviews, that including ECRs in the running of scientific societies brings benefits to both parties, and that there is considerable scope to increase the number of ECRs involved in these roles. Giving ECRs a voice in the decision-making processes for scientific societies may also encourage other organizations to provide them with a larger leadership platform. For instance, funding agencies such as the Canadian Institute of Health Research (CIHR) and the National Institutes of Health (NIH) have included ECRs on their Governing Council (Canadian Institute of Health Research, 2020), and the Working Group for the Advisory Committee to the Director (National Institutes of Health, 2018), respectively. Publishing groups and scientific journals have also started including ECRs on their leadership team, such as the Early Career Advisory Group set up by *eLife* (eLife, 2020). Given the critical role of ECRs in driving the diversity of research, offering ECRs a more prominent position in scientific societies will benefit the research enterprise in the long term.

## Data Availability

All data generated or analysed during this study are included in the manuscript and supporting files.

## Additional files

Supplementary file 1. Crowd-sourced data on ECR leadership at 20 scientific societies.

Supplementary file 2. List of survey questions sent to executives and ECRs in leadership positions within societies.
