# Supplementary material for: Why scientific societies should involve more early-career researchers
Source: eLife. 2020 Sep 23;9:e60829. doi: 10.7554/eLife.60829 (PMC7511228; doi:10.7554/eLife.60829)
Supplement: Supplementary file 2. [file elife-60829-supp2.docx]

### Supplementary file 2. List of survey questions sent to scientific society executives and ECRs in leadership positions within societies:

For society leaders/Presidents/EDs

- **Background on these positions:**
  - How long have you had ECRs in leadership positions within your society?
  - What type of positions are these? Are ECRs voting members?
  - Can you share any demographic data (career stage, gender, geography, race/ethnicity) about the ECRs in these positions?
  - Why was it important to add an ECR on the board for your society?
- **Experiences in these positions:**
  - What were the criteria for selecting the ECRs in these positions; what did you look for?
  - How long do ECRs serve in these positions and what are their responsibilities?
  - What have ECRs in your society been able to accomplish in these positions?
  - Have you surveyed their experience and whether these positions helped them in their careers?
- **Barriers and successes in these positions:**
  - Were there particular challenges *during the process* of adding an ECR to your board or other leadership position?
  - Who did the opposition come from (if any?) and how did you overcome this?
  - What has the society gained from having ECRs in these positions?
  - Did you find any drawbacks to having ECRs in these roles, and what would those be?
  - Are there any pointers from these experiences to pass on to other societies?

For ECR leaders

- **Background on these positions:**
  - How long have you served on the board and how long are these terms typically? Are you in a voting position? If yes, do you value that? If not, would you want that? If not on the board, do you hold a leadership position in the society?
  - Can you share any demographic data (career stage, gender, geography, race/ethnicity) about yourself and/or other ECRs in these positions?
  - What value did this position bring to you professionally?
- **Experiences in these positions:**
  - What are qualities of successful ECR board members within this society?
  - What have your responsibilities been in your position?
  - How have you contributed to the mission and success of the organization from this position?
  - Has your experience in this position been positive or negative, and why?
- **Barriers and successes in these positions:**
  - How has serving as an ECR leader impacted your career?
  - Are you the only ECR leader in this position, or are there others, and how did that impact you?
  - Have you encountered struggles to making your voice heard among leadership?
  - Are there any pointers from these experiences to pass on to others who want to join?
